# Exploiting Sequence-Dependent Rotamer Information in Global Optimization of Proteins

**DOI:** 10.1021/acs.jpcb.2c04647

**Published:** 2022-10-18

**Authors:** L. Dicks, D. J. Wales

**Affiliations:** † Yusuf Hamied Department of Chemistry, University of Cambridge, Lensfield Road, Cambridge CB2 1EW, United Kingdom; ‡ IBM Research, The Hartree Centre STFC Laboratory, Sci-Tech Daresbury, Warrington WA4 4AD, United Kingdom

## Abstract

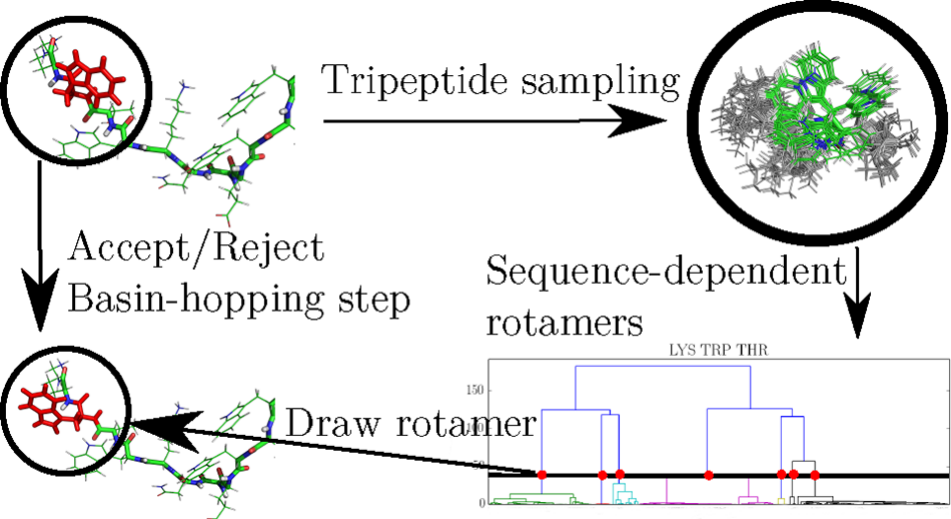

Rotamers, namely amino acid side chain conformations common to many different peptides, can be compiled into libraries. These rotamer libraries are used in protein modeling, where the limited conformational space occupied by amino acid side chains is exploited. Here, we construct a sequence-dependent rotamer library from simulations of all possible tripeptides, which provides rotameric states dependent on adjacent amino acids. We observe significant sensitivity of rotamer populations to sequence and find that the library is successful in locating side chain conformations present in crystal structures. The library is designed for applications with basin-hopping global optimization, where we use it to propose moves in conformational space. The addition of rotamer moves significantly increases the efficiency of protein structure prediction within this framework, and we determine parameters to optimize efficiency.

## I Introduction

Early in protein structural studies, it was observed that amino acid side chains occupy a relatively small number of conformations, which are identifiable in many different peptides.^1^ Consequently, efforts began to characterize the side chain conformations common to each amino acid, known as rotamers (rotational isomers). Rotamers are classified by a list of the dihedral angles present in the particular side chain conformation. Bond lengths and angles are omitted as they are assumed to be approximately ideal in all rotamers. Each amino acid supports its own set of rotamers, and the complete set, for all amino acids, can be tabulated in libraries.^2^ A rotamer entry usually specifies the amino acid, the dihedral angles, with an associated measure of variance, and the probability of occurrence.^3^ Many rotamer libraries have been constructed and have been used in applications such as crystallographic model building,^4−7^ protein–ligand docking,^8−12^ homology modeling,^13−16^ and protein design.^17−23^ Within these applications, it is also possible to use machine learning to predict the most probable rotamer for a given conformation.^24−26^ Moreover, the native structure of many proteins can now be predicted at atomic accuracy by neural networks,^27^ but there remain numerous peptide classes with little experimental data and important cases where we require additional minima beyond the native conformation. One conformation is insufficient for sampling the thermodynamic properties of the folding funnel and for predicting competing conformations and their transition rates. Hence, there are applications where rotamer libraries are likely to be useful.

Many rotamer libraries are derived experimentally from data available in the protein data bank (PDB).^28^ In each case the curation of a representative set of protein structures, from which to extract rotamers, is the main consideration in the construction of the library. However, limitations in the ability of rotamers derived from crystal structures to reflect conformations in solution have been highlighted.^29,30^ Side chains are sensitive to the crystal environment,^31,32^ unique side chain rotamers can occur as a result of cryo-cooling,^33^ and side chain detail can be absent.^34^ The generation of rotamer libraries from computer simulations has therefore been explored. Molecular dynamics (MD) simulations of many distinct proteins folds were used to generate the dynameomics rotamer library,^35,36^ which contains dynamic information about side chain motion, absent from static crystal structures. The simulations generated significantly more relevant side chain conformational data than experiment, and some insight into rotamer dynamics can be extracted.^37^ Moreover, simulations have been used to obtain side chain information about systems for which experimental data was not available.^38^

Rotamer libraries can be partitioned into categories depending on the information they encode, the most common of which are backbone-independent,^35,39−41^ backbone-dependent,^42−44^ and secondary structure-dependent libraries.^45,46^ Backbone-independent rotamer libraries are constructed such that amino acid side chain conformations are averaged over the possible backbone dihedral angles. In contrast, in the latter two libraries the rotamers and their probabilities are modulated by either the ϕ, ψ dihedral angles or the secondary structure of the corresponding amino acid. The relative success of these different categories has been recently assessed.^47^

Efforts have been made to construct databases accounting for additional factors that influence rotamer populations, leading to the development of protein-dependent,^48,49^ position-specific,^50^ and sequence-dependent rotamer libraries.^51^ Sequence-dependent libraries assume that the observed rotamers of a side chain are largely controlled by interactions with adjacent amino acids and, therefore, contain a distinct set of rotamers for every possible sequence. Rotamer libraries have also been established for improved modeling accuracy of specific systems, such as peptoid foldamers,^38,52^ coarse-grained peptides,^53^ and antibodies.^54^

In this contribution we constructed a sequence-dependent, backbone-independent rotamer library from simulations of all possible tripeptides composed from naturally occurring amino acids. Basin-hopping global optimization^55,56^ was used to find low-energy conformations of each tripeptide, and from the resulting conformations the rotamers of each central amino acid were extracted for all possible adjacent residues. The resulting library was used to propose moves in conformational space for basin-hopping global optimization, which improves the efficiency significantly over current basin-hopping schemes based on dihedral rotations. Both the rotamer library and links to the software used throughout this work are provided in the Supporting Information.

## II Methods

### II.A Tripeptide Conformations

Sequence-dependent rotamer libraries (SDRLs) include a specific set of amino acid rotamers for all possible combinations of adjacent residues. Therefore, construction of an SDRL requires stable peptide conformations for every sequence. In our computational methodology, we constructed all possible tripeptides composed of the 18 naturally occurring amino acids, aside from alanine or glycine, as a central residue, including the three distinct protonation states of histidine. Alanine and glycine were excluded as the central residue because their side chains are too simple to support rotameric states. Proline was excluded owing to the presence of only two side chain conformations. Each tripeptide was capped by an acetyl group and a methylamide group at the C- and N-termini, respectively, giving tripeptides of the form ACE–XXX–YYY–ZZZ–NME.

The global and low-energy minima of each tripeptide were located using basin-hopping (BH).^55,56^ Basin-hopping is a global optimization algorithm that searches potential energy surfaces (PESs) transformed into basins of attraction according to
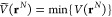
1*V*(**r**^*N*^) is the potential energy, **r**^*N*^ is the 3*N*-dimensional vector corresponding to a point in the configuration space, and min{*V*(**r**^*N*^)} denotes the potential energy obtained after local minimization, starting at **r**^*N*^. Local minimization of each point in space was performed using the limited-memory Broyden–Fletcher–Goldfarb–Shanno (LBFGS) algorithm.^57,58^ The transformed PES was explored by generating new configurations using geometric perturbations, then minimizing, and accepting or rejecting the new minimum based on a Metropolis-like criterion.^59^

A basin-hopping scheme that applies an acceptance criterion for new minima based on their local free energies, free energy basin-hopping (FEBH),^60,61^ was used here. The local free energy of each encountered minimum, estimated using the harmonic superposition approximation,^62−65^ was calculated, and the Metropolis-like acceptance criterion was applied to free, rather than potential, energy differences. The corresponding potential energy minimum was also stored.

Fifty thousand FEBH steps were performed for each tripeptide with structural perturbations achieved using group rotation moves^66,67^ and random atomic displacements of up to 1 Å. Group rotation moves stochastically select ϕ, ψ, and χ dihedrals and apply a rotation of a randomly selected magnitude. These moves were attempted every two FEBH steps, and the probability of selecting any dihedral was set to 0.025. Potential energies of minima were evaluated using the properly symmetrized^68^ AMBER^69,70^ ff14SB force field,^71^ which was selected to provide good accuracy at low computational cost. However, the choice of force field can bias the sampling of conformational states,^72−75^ and several studies have calculated energies of tripeptides using significantly more expensive quantum chemistry methods to reduce this bias.^76,77^ Solvent water was modeled implicitly within a generalized Born framework,^78,79^ and a salt concentration of 0.1 M was included using the Debye–Hückel approximation.^80^ The generalized Born framework is a fast, approximate representation of a solvent that captures the dielectric shielding of electrostatics but the absence of explicit water molecules affects the solvent–solute dispersion representations and the effect of tightly bound water molecules. For each tripeptide, the low-energy conformations contain many ϕ, ψ combinations, and the observed rotamers of the central amino acid are averaged over these backbone configurations, resulting in a backbone-independent library.

In contrast to experimental libraries, we estimate rotameric occurrence probabilities from approximate conformational free energies. Each tripeptide conformation was assigned its equilibrium population at 298 K as the probability of occurrence. The free energy of each complete tripeptide was used, so the probability of observing a rotamer of the central amino acid side chain explicitly includes energetic contributions accounting for the strain, in adjacent residues and the backbone, to accommodate the central side chain conformation.

Using tripeptide structures allows local spatial effects on rotamers to be probed without interference from stabilization in protein folds. Consequently, the library is constructed from conformations that may be more relevant in exposed surface residues, rather than the predominantly buried environment used in experimental libraries. Surface side chains cannot support high-energy rotamers that are stabilized by nonlocal effects, as in protein folds, but show greater conformational flexibility owing to reduced steric effects.^81−83^ This flexibility should allow us to capture all the relevant rotamers for both surface and interior residues.

### II.B Clustering

**Figure 1:**
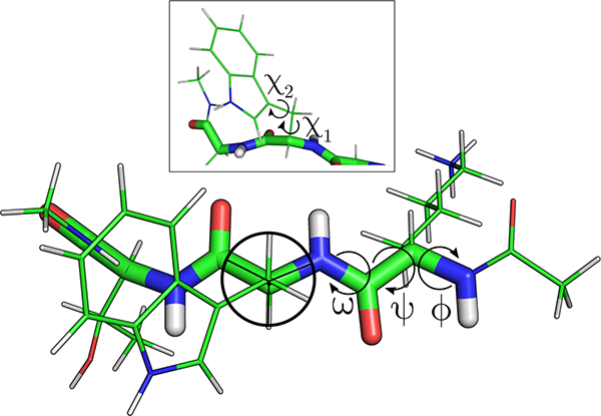
An example capped tripeptide with the ϕ, ψ, and ω backbone dihedrals and the χ side chain dihedrals highlighted. The boundaries between rotameric bins at 0°, 120° , and −120° are shown for the tryptophan χ_1_ dihedral.

Extraction of rotamers from peptide structures is usually performed by either clustering^38,84−86^ or binning.^2^ Binning specifies possible angle ranges (bins) in which distinct rotamers can exist, based on the central bond of each χ dihedral. Conformations in different bins are considered distinct, as they are separated by large energetic barriers corresponding to a transition state with eclipsed bonds.^87^ Binning is performed for each dihedral in a side chain, and the average angles within each bin are calculated to determine the corresponding rotamer. For sp^3^–sp^3^ bonds, bins are centered about the staggered trans and gauche conformations with boundaries defined at 0°, −120°, and 120°, shown in Figure 1. sp^3^–sp^2^ bonds have more complex, broader, and more asymmetric rotameric distributions,^41^ so suitable bin definitions are not obvious, and alternative formulations have been used.^51^

Here, the clustering approach was preferred because it does not exclude the possibility of multiple rotamers within a single bin and avoids the problem of bin definition for sp^3^–sp^2^ bonds. Hierarchical average-linkage agglomerative clustering^88^ was performed in torsional space, and the average conformation within each cluster was assigned to a distinct rotamer. Hierarchical clustering allows for a variable number of clusters that satisfy the condition for rotamericity.

We chose to cluster in torsional, rather than Euclidean, space to preserve the rotational energy barriers for each of the χ bonds. When conformations are compared in Euclidean space, less importance is placed on χ dihedral angles as the number of bonds separating them from the peptide backbone increases. This bias may lead to grouping of distinct rotamers that differ only in the final χ angle, which would still be separated by a significant energetic barrier.

The distance metric, chosen to measure the dihedral angle similarity between side chain conformations, was the Euclidean distance between side chain conformations in torsional space
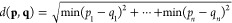
2*n* is the number of dihedral angles in the side chain. **p** and **q** are *n*-dimensional vectors of conformations in torsional space. min specifies calculation of the minimum distance between any two angles, accounting for periodicity, e.g., 1° between −179° and 180°.

**Figure 2:**
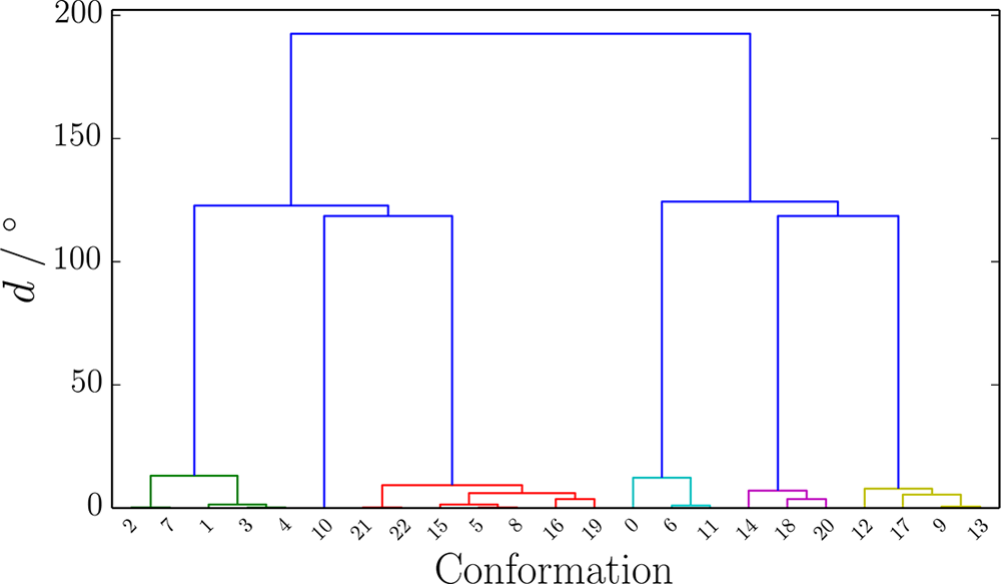
Dendrogram generated by agglomerative hierarchical clustering of the central amino acid side chain conformations for the ALA–PHE–ALA tripeptide. Each conformation is listed on the horizontal axis, and their interrelations are given by the height at which the two conformations merge on the vertical axis, which gives the value of our distance metric. A 40° cutoff was used to separate clusters in all tripeptides; here, this cutoff produces six clusters.

A value of 40° in the distance metric, *d*, was chosen to separate each dendrogram into flat clusters, each of which corresponds to a single rotamer. Dendrograms display the hierarchical composition of clusters through merging of vertical lines corresponding to side chain conformations, and an example dendrogram is given in Figure 2. This condition closely resembles a metric that defines rotamers as the same if they differ by less than 40° in each χ angle.^89^ However, our condition is stricter because, in addition to rotamers being closer than 40° in any dihedral, their average Euclidean distance in torsional space must also be less than 40°.

For each cluster, we calculated the mean of each separate χ angle in each member conformation. Statistical modes, although providing a better representation of skewed angle distributions, were not used because of the small data sets and the strict clustering condition. Clustering is expected to mitigate some previous problems with significantly non-Gaussian distributions, where multiple distinct rotamers within a bin were merged. The corresponding standard deviation was calculated, and for clusters containing only a single conformation we assigned a standard deviation of 1.0° to account for possible variance arising from displacements about the corresponding minimum. The occupation probability of each conformation at 298 K belonging to the cluster was added to give its total probability. The clustered conformations were compiled into the resulting sequence-dependent rotamer library by removing those that have an occupation probability of less than 0.005.

### II.C Basin-Hopping

The constructed rotamer library was used to implement new basin-hopping schemes for peptides, with alternative trial moves applied to side chains. We compared the efficiency with our current schemes that apply group rotation moves to both randomly selected peptide backbone and side chain dihedrals. The proposed rotamer schemes limit group rotation moves to peptide backbone dihedrals and apply rotamer moves that impose rotameric conformations on side chains. Amino acids were selected uniformly, but each rotamer was selected with its corresponding occupation probability.

A variety of rotameric schemes were tested to determine optimal parameters for global optimization of peptide sequences. The parameters varied were the frequency and number of rotamer moves and backbone dihedral rotations, producing 12 combinations. No random atomic displacements were applied, and basin-hopping was performed at a fixed sampling temperature of *T* = 1.3 kcal mol^–1^. This temperature parameter controls the acceptance of new states via a Metropolis accept/reject type scheme, and it is usually expressed in energy units.

The efficiency of each scheme was compared for its location of the global potential energy minimum conformations of a tryptophan zipper (PDB code: 1LE0)^90^ and the dimer of the short amyloidogenic peptide sequence KFFE. The 12 rotamer schemes were compared with four alternatives that apply group rotation moves to both backbone and side chain dihedrals. These relatively small systems were employed for benchmarking purposes. Our aim was to determine parameters that will hopefully be effective for larger systems of practical interest.

## III Results and Discussion

### III.A Library Analysis

To evaluate the quality of the tripeptide conformational sampling, we compared our backbone-independent rotamer library to experimental crystal structures and the most widely used example in this class: the penultimate rotamer library.^39^ The penultimate library is not sequence-dependent, so in our comparisons we considered averages over all sequences for each amino acid. This approach should give a fair comparison, as the penultimate library was constructed from structures containing amino acids in many different peptide sequences. However, we averaged over all possible sequences equally, which is not the case in the experimental library, where preference was given to certain triplets based on their occurrence frequency in the protein structures used to compile the library.

For the following analysis, we distinguish the clustered tripeptide side chain conformations from the rotamer library. The rotamer library constitutes the subset of conformations with an occupation probability of greater than 0.005. Despite the short chain length, the distribution of backbone dihedral angles is comparable to results for full-length proteins, as shown in the Ramachandran plots in Figure S1. Moreover, the clustered conformations for each central side chain include all rotamers of the penultimate library.

The complete clustered side chain data has a significant proportion of sequences that exhibit all penultimate rotamers, 51.1%, which is reduced to 36.0% when excluding side chains with only one χ dihedral. The absence of some penultimate rotamers in many sequences highlights the constraints of local sequence and the possibility of using this information to reduce the side chain search space significantly. When limited to sequence-dependent rotamers, only 3.2% of sequences contain all the penultimate rotamers for side chains with more than one χ dihedral. Hence, the sequence-dependent rotamers should provide a more compact subset for each sequence.

**Figure 3:**
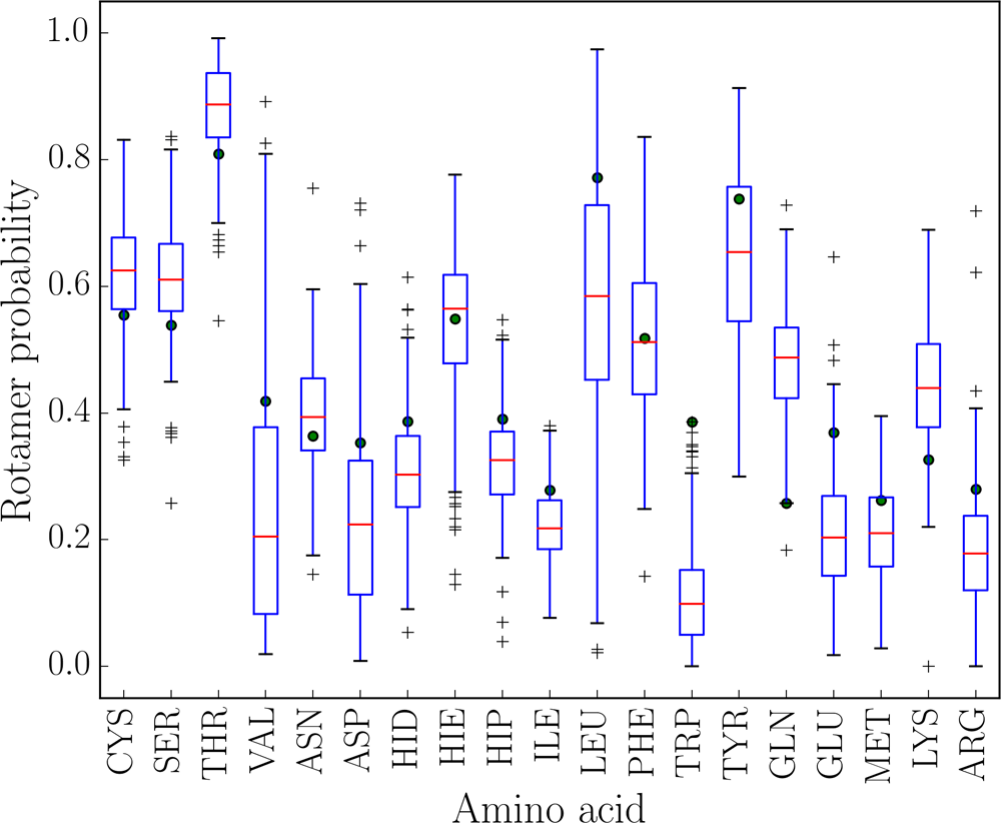
Box plot showing the variation in the probability of a particular rotamer with sequence. The box extends from the first quartile to the third quartile of the data, with a red line at the median. The whiskers extend to 1.5 times the interquartile range, and values outside whiskers are indicated by crosses. The most probable rotamer in the GLY–XXX–GLY tripeptide was used as the reference, as this is the most favorable rotamer for the unhindered side chain, having no steric interactions from adjacent glycines. Green circles indicate the probability of the rotamer in GLY–XXX–GLY. The plot shows that rotamers can be significantly promoted or suppressed by sequence.

The subset of penultimate rotamers for each central amino acid results from modified rotamer probabilities with sequence, Figure 3. As an example, the most probable rotamer in each GLY–XXX–GLY tripeptide was located and its probability monitored, where XXX was replaced by each amino acid. This rotamer was considered a suitable reference to monitor sequence variation because the adjacent glycines place minimal constraints on the central amino acid side chain, so it closely approximates the most energetically favorable conformation of the unconstrained side chain. This rotamer was then identified in all other tripeptides with the same central amino acid, using the 40° metric discussed for agglomerative clustering.

The probability of the most favorable unhindered rotamer changes significantly for all amino acids, demonstrating that the local sequence exerts important steric constraints on side chains. Side chains with fewer than three χ dihedrals exhibit greater variation than those with more dihedrals for two reasons. First, the sharp increase in the number of rotamers at three χ dihedrals means that each rotamer has a reduced probability. Second, side chains with fewer than three χ dihedrals have little flexibility to relieve steric clashes, leading to much more significant fluctuations in energy and, therefore, probability. This effect was also seen in previous work, where the order of preference for rotamers was analyzed and a large variation with sequence was found.^51^

To further validate our computationally derived SDRL, we tested the number of experimentally observed side chain conformations present in the library. The chosen experimental data was the set used to compile the penultimate rotamer library.^39^ The data set contains 500 crystal structures filtered from the PDB for high quality and resolution. The penultimate library, derived from the same data, contained 94.5% of the experimental side chain conformations; 5.5% were not assigned, as the rotamer library was restricted to contain only the most common side chain conformations.

Sequence information was extracted from PDB files, and side chain conformations were removed when the sequence was not available or contained amino acids not present in our library. The condition for matching side chain conformations was the same as that used in the evaluation of the penultimate rotamer library, where a correct assignment must lie within 40° in each χ dihedral. We first match to the successful penultimate rotamer, if present, and if not we consider a direct match to the experimental data. For histidine the rotamers of the δ protonation state were used, as the protonation state was not extracted, and this choice gives the most effective representation of the experimental data.

The clustered side chain conformations retain a very high percentage of the experimental states, as shown in Table 1. The number of such structures is naturally much higher than in the penultimate library, which clusters all the stable tripeptide conformations supported by the force field. However, the successful reproduction of experimental data within each sequence provides evidence for the effectiveness of modeling with tripeptides.

**Table 1:** Percentage of Experimental Side Chain Conformations That Are Present in Rotamer Libraries for the Training Data of the Penultimate Rotamer Library^a^

amino acid	complete data/%	rotamers/%	penultimate/%
CYS	99.6 (3.0)	99.4 (3.0)	99.0 (3)
SER	99.6 (3.0)	99.6 (3.0)	98.6 (3)
THR	99.9 (3.0)	99.7 (2.9)	99.6 (3)
VAL	99.6 (3.0)	99.6 (3.0)	99.2 (3)
ASN	96.7 (13.6)	88.6 (7.8)	92.5 (7)
ASP	96.1 (6.3)	93.9 (5.0)	89.4 (5)
HIS	87.4 (9.8)	86.1 (8.0)	90.2 (8)
ILE	99.5 (9.5)	99.1 (7.3)	91.9 (7)
LEU	99.4 (9.5)	98.0 (5.0)	96.6 (5)
PHE	92.1 (3.1)	92.1 (3.0)	98 (4)
TRP	93.0 (8.1)	92.3 (6.0)	95.9 (7)
TYR	93.6 (3.2)	93.5 (3.0)	98 (4)
GLN	85.1 (25.9)	83.5 (12.7)	81.3 (9)
GLU	88.5 (16.1)	72.7 (7.2)	82.3 (8)
MET	96.6 (25.5)	81.6 (15.7)	85.1 (13)
LYS	92.5 (40.6)	75.0 (12.6)	78.1 (27)
ARG	91.9 (77.0)	57.0 (20.3)	83.3 (34)
total	95.8	91.4	94.5

a The penultimate library performance was used as a reference. Complete data uses all clustered conformations for each sequence, whereas rotamers compare to only the most populated conformations. The average number of side chain rotamers (or conformations) across all sequences is given in parentheses.

Pruning conformations into rotamers degrades the performance in reproducing experimental data, as expected when removing conformational states. However, the reranking of conformations based on their occupation probability retains 91.4% of the experimental data. For the experimental data not successfully assigned by the rotamer library, the average distance to the correct assignment was 16.5 ± 9.5°, indicating many of these experimental conformations are close to successful assignment. The performance is slightly lower than that of the penultimate library for a comparable number of rotamers, which is expected for rotamers derived from occupation probabilities of surface side chains at 298 K. However, the rotamer library derived with this methodology still captures many of the low-temperature side chain conformations within protein folds. Furthermore, rotamers derived in this manner are valuable for global optimization, where the ability to represent the conformational freedom of both surface and buried side chains at room temperature is essential. Moreover, the effect of protein folds will be automatically compensated for in basin-hopping using local minimizations.

### III.B Basin-Hopping Schemes

We now exploit the rotamer library to propose conformational perturbations in basin-hopping global optimization. We compare a variety of different schemes, given in Table 2, that are distinguished by applying either rotamer or group rotation moves to side chains when proposing new candidate peptide conformations. Backbone dihedrals are modified by group rotation in both cases to allow comparison of the effect of the side chain conformation. A variety of different schemes can be constructed using rotamer and group rotation moves, changing the number and frequency of backbone and side chain perturbations, and we were guided by previous successful applications to proteins.^74,91^ We applied these formulations to both the tryptophan zipper and the KFFE dimer.

**Table 2:** Comparison of Rotamer and Group Rotation Schemes in Basin-Hopping Global Optimization^a^

scheme	*n*_SC_	*f*_SC_	*n*_BB_	*f*_BB_
rotamer 1	2	1	1	1
rotamer 2	2	1	2	2
rotamer 3	3	1	1	1
rotamer 4	2	1	3	3
rotamer 5	2	2	2	2
rotamer 6	2	1	3	4
rotamer 7	3	1	3	3
rotamer 8	3	2	2	2
group rotation 1	4	1	1	1
group rotation 2	4	1	2	2
group rotation 3	6	1	1	1
group rotation 4	6	1	2	1

a The moves are defined by the number of backbone dihedrals, *n*_BB_, changed and the number of BH steps between backbone moves, *f*_BB_. Side chains are perturbed every *f*_SC_ steps, by either rotamer moves or group rotation, and the number, *n*_SC_, corresponds to either the number of selected side chains or the number of side chain dihedrals, respectively.

Schemes 1, 2, and 3 were designed to compare rotamer and group rotation moves, with the total number of side chain dihedral changes similar for both peptides. The first three schemes therefore permit a direct comparison of the efficiency of rotamer moves and uncorrelated dihedral rotations.

The tryptophan zipper was optimized starting from a linear chain of amino acids, and the KFFE dimer was optimized from a parallel β-sheet arrangement. The global minimum of the dimer is an antiparallel β-sheet, with several alternative conformations that produce distinct free energy funnels.^92−94^ The starting points were chosen to make global optimization more challenging.

Basin-hopping was run for a fixed number of steps, 400 000 and 50 000, for the tryptophan zipper and KFFE dimer, respectively. We performed three basin-hopping runs for each set of moves and considered runs to be successful if they encountered a structure within 1 kcal mol^–1^ of the global minimum. Structures within this energy range are very similar to the global minimum. The RMSD between successful structures within this energy range and the global minimum is 0.09 Å for the tryptophan zipper and 2.05 Å for the KFFE dimer, which is larger because of the greater flexibility of two peptide chains.

#### III.B.1 Rotamer vs Group Rotation

Basin-hopping schemes involving rotamer moves applied to side chains, rather than group rotations, provide a marked improvement for global optimization in both the tryptophan zipper and the KFFE dimer (Table 3). The improvement is observed for almost all rotameric schemes. We see only a single successful basin-hopping run with group rotation in the allotted number of steps, whereas only one rotamer scheme does not achieve at least two successful runs. The lower success rate for the KFFE dimer is due to the reduced number of steps, which was chosen to limit the computational cost.

**Table 3:** Performance of Different Rotamer and Group Rotation (GR) Schemes in Global Optimization of the Tryptophan Zipper and KFFE Dimer^a^

	tryptophan zipper			KFFE dimer		
scheme	successes	*n*_steps_/1000	Δ*E*	successes	*n*_steps_/1000	Δ*E*
rotamer 1	1	142.1	2.91	1	10.5	1.78
rotamer 2	3	61.6		2	9.6	1.40
rotamer 3	0		3.98	0		1.32
rotamer 4	2	182.0	4.92	0		1.62
rotamer 5	1	30.4	4.66	1	4.6	1.79
rotamer 6	2	75.9	1.11	1	11.8	1.83
rotamer 7	2	157.7	4.99	0		1.33
rotamer 8	1	219.2	4.74	1	46.9	1.78
GR 1	0		6.33	0		1.19
GR 2	0		7.77	0		1.50
GR 3	0		4.98	0		1.78
GR 4	1	173.0	6.14	0		1.82

a A basin-hopping run was considered successful if it encountered a structure within 1 kcal mol^–1^ of the global minimum within 400 000 or 50 000 basin-hopping steps for the tryptophan zipper or the KFFE dimer, respectively. Δ*E* provides the average energy above the global minimum for the unsuccessful global optimizations in kcal mol^–1^. *n*_steps_ is the number of basin-hopping steps required to find the global minimum for successful basin-hopping runs.

The rotamer schemes exhibit good performance for a range of parameters. Scheme 2 is the most efficient, and these are the parameters we recommend for basin-hopping analysis of novel peptides. The most successful schemes involve perturbing a relatively small number of side chains at every BH step, which leads to the efficient location of stable side chain packings for each backbone configuration.

For the tryptophan zipper, we note that the structures encountered in the unsuccessful group rotation runs are much higher in energy than in the rotamer schemes, and these runs did not come close to locating the global minimum. For the KFFE dimer, the group rotation schemes produce structures much closer to the global minimum, indicating that despite the lack of successful runs, the relative performance is not so bad.

Efficient side chain moves allow the peptide backbone to explore low-energy conformations, with side chains rapidly converting between stable conformations, providing good solutions to the side chain packing problem at each backbone configuration. It is challenging to identify the optimal side chain packing by direct enumeration, even for small systems, because of the combinatorial possibilities.^95,96^ However, several deterministic methods have shown it is possible to locate good solutions in polynomial time.^97−100^ Moreover, with increasing evidence for multiple stable packings of side chains,^101,102^ we need only find a good, rather than optimal, packing for each backbone configuration encountered to assess its stability.

Backbone displacements are essential to achieve side chain packing rearrangements.^103,104^ Our schemes explicitly account for the interplay between backbone and side chains through iterative changes to backbone dihedrals, followed by side chain conformations, with local minimization allowing both backbone and side chains to adapt their conformations. Furthermore, the local minimization performed in BH guarantees that we find the true rotameric structure for the given protein environment, defined as a local minimum on the dihedral PES.^105^

**Figure 4:**
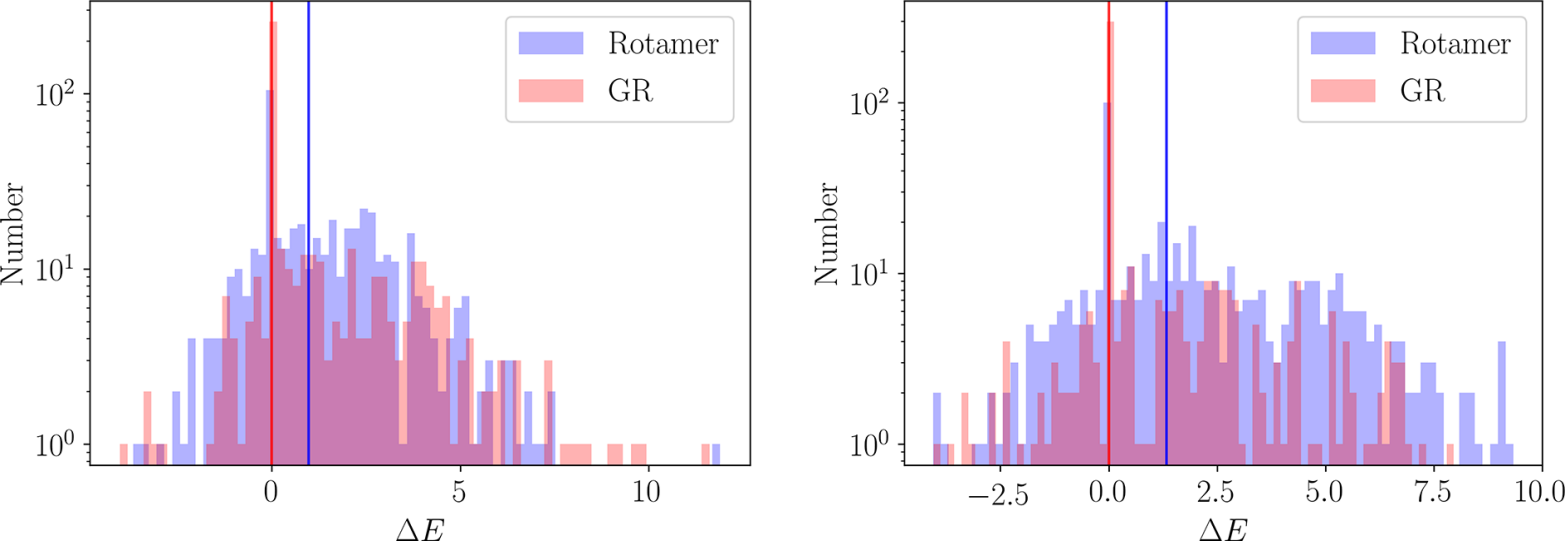
Difference in energy at each basin-hopping step, Δ*E*, measured relative to the current minimum in the Markov chain. The differences are calculated for a basin-hopping run with scheme 2 for both the tryptophan zipper (left) and the KFFE dimer (right). The median energy difference is denoted by a solid vertical line.

The improved efficiency of the rotamer schemes results from better sampling of the stable side chain packings at each backbone configuration. We observe that the rotamer moves after local minimization produce structures with a slightly higher relative energy, Δ*E*, than the corresponding group rotation schemes, Figure 4. The energy difference is measured from the proposed minimum to the current minimum in the Markov chain. The distribution of energies is similar in group rotation and rotamer schemes, but for the rotamer moves the results are skewed to slightly higher values, producing a larger median energy.

**Figure 5:**
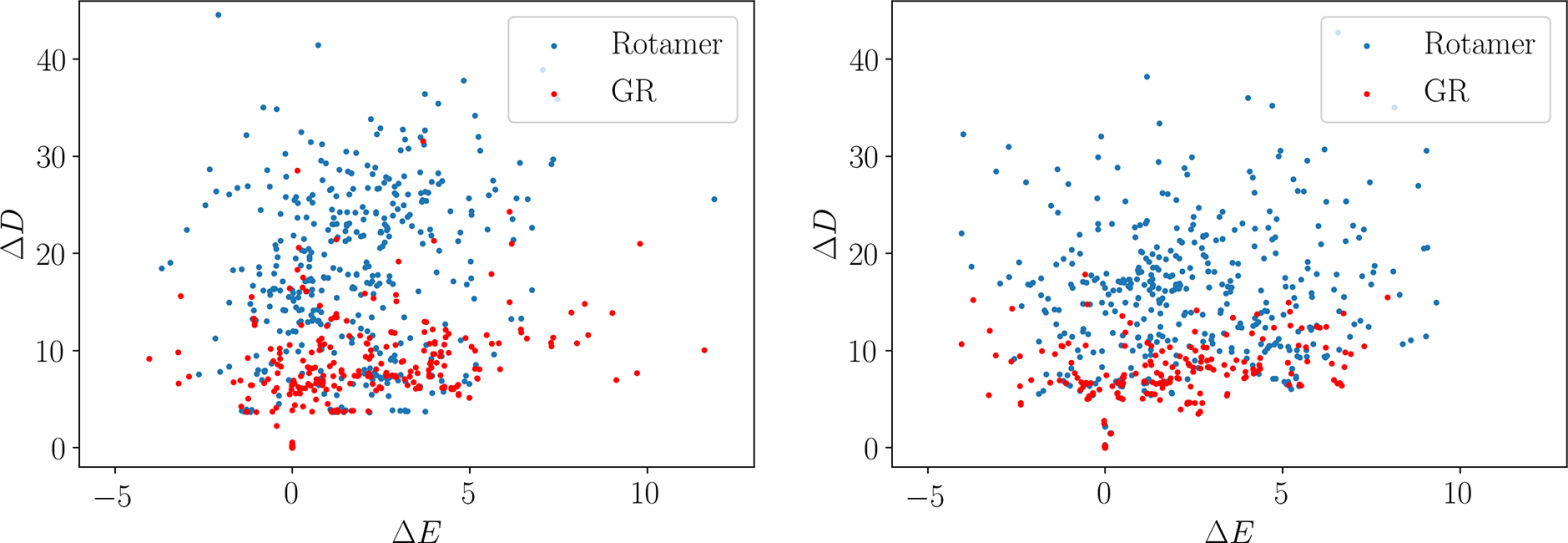
Change in energy and distance between adjacent structures in the accepted sequence of minima during a basin-hopping run. Δ*D* is given in Å, and Δ*E* in kcal mol^–1^. Results are presented for scheme 2 for both the tryptophan zipper (left) and the KFFE dimer (right).

The small energy difference between the schemes indicates that they produce peptide conformations of similar stability. However, the rotamer schemes allow larger perturbations to be applied to the side chains, while producing candidate structures of similar quality (Figure 5). The use of rotamers therefore allows more diverse candidate structures to be proposed, allowing faster sampling of the side chain packings. Equivalent plots are provided for scheme 1 in Figures S2 and S3.

Another essential component of the computational cost of basin-hopping is the number of potential energy evaluations required for each local minimization. Conformations further from their corresponding local minimum will likely require more LBFGS steps to locate the local minimum. The average number of evaluations needed for minimization at each basin-hopping step is shown in Table 4.

**Table 4:** Average Number of LBFGS Steps during a Local Minimization^a^

	trypzip		KFFE dimer	
scheme	*n*_BB–SC_	*n*_SC_	*n*_BB–SC_	*n*_SC_
rotamer 1	934	869	1028	945
GR 1	1075	1028	1049	1000
rotamer 2	939	855	1186	1104
GR 2	1050	1032	1061	984

a The RMS force convergence criterion was 10^–6^ kcal mol^–1^ Å^–1^. BH moves involving rotation of backbone dihedrals are separated from moves that only perturb side chain conformations.

We see that for the tryptophan zipper both rotamer schemes require significantly fewer steps to attain the same accuracy for each local minimization. Despite the larger perturbations when proposing candidate structures, the rotamer moves, using a local minimum of the side chain in a tripeptide, require less computation for reoptimization. The number of minimization steps at each basin-hopping step in the rotamer schemes is around 80% of the steps required in the corresponding group rotation schemes, providing further computational gains. A similar result is seen for the KFFE dimer in scheme 1; however, this trend is reversed for scheme 2.

## IV Conclusions

We have developed a methodology for the construction of rotamer libraries using basin-hopping global optimization of tripeptides. The library is derived without reference to experimental data and, because of the short peptide chains, without the influence of protein folds. The use of tripeptides allows the effect of sequence to be included in this library, which captures 91.4% of the low-temperature experimental side chain data within the protein folds considered. The rotamers can be used efficiently in global optimization, as they provide a room-temperature representation of surface side chains under the local influence of sequence.

Applying this sequence-dependent rotamer library in basin-hopping schemes provides a significant improvement over previous results for the global optimization of peptide sequences in our benchmarks. The use of rotamer moves, coupled with group rotation moves for the backbone, searches the side chain space efficiently, while adapting to backbone rearrangements. The rotamer moves allow much larger perturbations, while still producing relevant candidate structures.

The increased efficiency in the number of steps needed to locate the global minimum is profound. Furthermore, the rotamer schemes generally require a smaller number of minimization steps to reach local minima. Hence, the advantage of rotamer moves in basin-hopping is twofold, requiring fewer energy and gradient evaluations at each basin-hopping step, while also reducing the number of basin-hopping steps needed to locate the global minimum.

## Notes

The data that support the findings of this study are available within this article and its Supporting Information.

## Supporting Information Available

Rotamer library (ZIP)
Tripeptide conformations (ZIP)
Ramachandran density plots for the tripeptide conformations; figures for basin-hopping schemes; code repository used in this work, along with the corresponding library (PDF)
