# Supplementary material for: Exploiting Sequence-Dependent Rotamer Information in Global Optimization of Proteins
Source: J Phys Chem B. 2022 Oct 18;126(42):8381–90. doi: 10.1021/acs.jpcb.2c04647 (PMC9623586; doi:10.1021/acs.jpcb.2c04647)
Supplement: Supplementary file 3 — jp2c04647_si_003.pdf [file jp2c04647_si_003.pdf]

Supporting Information for Publication: Exploiting Sequence-dependent Rotamer Information in Global Optimisation of Proteins

L. Dicks† and D. J. Wales*

Yusuf Hamied Department of Chemistry, Lensfield Road,

Cambridge CB2 1EW, United Kingdom

(Dated: September 22, 2022)

† Author now affiliated with IBM Research Europe, Hartree Centre, Sci-Tech Daresbury, United Kingdom

* Corresponding author. Email: dw34@cam.ac.uk

I. ROTAMER LIBRARY

The minima for each tripeptide are given in the TripeptideConformations.tar.gz, which contains an associated README that explains the file format. The library itself, including all clustered side chain conformations, is given in Library.tar.gz, again with a description in the README file.

The Ramachandran density plots for the tripeptide conformations, averaged over all neighbouring amino acids, are given for each central amino acid in Fig. S1.

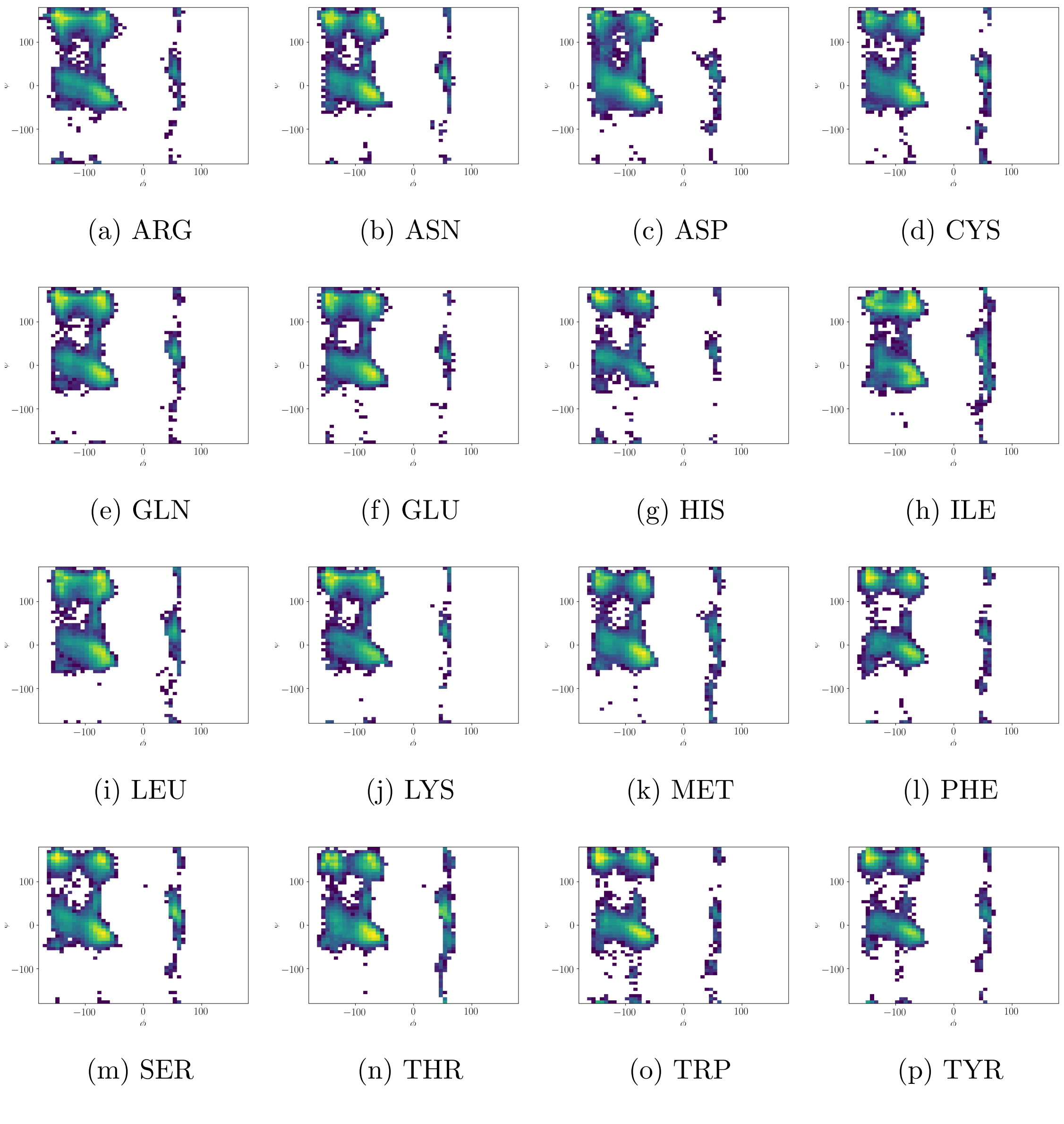

(a) ARG (b) ASN (c) ASP (d) CYS

(e) GLN (f) GLU (g) HIS (h) ILE

(i) LEU (j) LYS (k) MET (l) PHE

(m) SER (n) THR (o) TRP (p) TYR

FIG. S1: Ramachandran plots calculated for all minima of tripeptides corresponding to a single central side chain.

II. BASIN-HOPPING SCHEMES

The energy of the proposed candidate structures, after minimisation, in basin-hopping are shown in Fig. S2. The corresponding distance between proposed structures is given in Fig. S3. Both the figures are calculated for group rotation and rotamer scheme 1. The results show similar trends to those presented for Scheme 2 in the main text. The rotamer moves propose structures of a slightly higher energy, but with much larger perturbations.

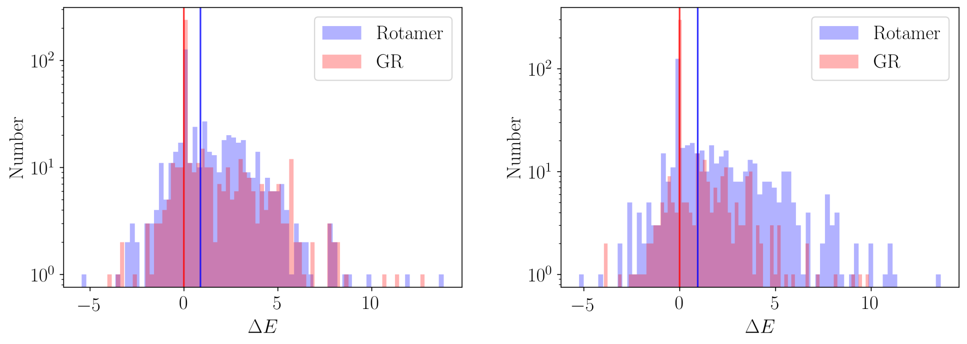

FIG. S2: The difference in energy at each basin-hopping step (ΔE), as measured relative to the current minimum in the Markov chain, for basin-hopping of the tryptophan zipper (left) and the KFFE dimer (right). Distributions are calculated for basin-hopping runs of group rotation and rotamer scheme 1. The median ΔE is given by a solid vertical line.

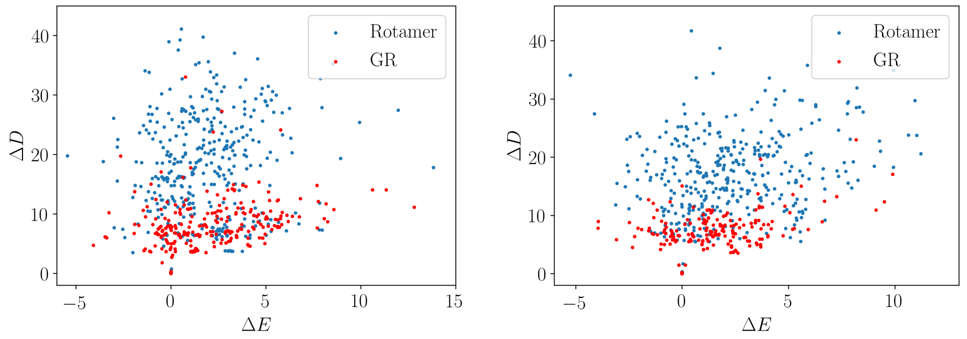

FIG. S3: The distribution of change in energy and distance between adjacent structures in the accepted sequence of minima during a basin-hopping run for the tryptophan zipper (left) and the KFFE dimer (right). ΔD is given in Å, and ΔE in $\mathrm{kcal\,mol^{-1}}$. Values are calculated for basin-hopping runs of scheme 1.

III. CODE REPOSITORY

The basin-hopping schemes presented are implented in the GMIN software, freely available at https://www-wales.ch.cam.ac.uk/GMIN/. A tutorial for GMIN is provided at https://github.com/wales-group/examples, with input keywords explained at https://www-wales.ch.cam.ac.uk/GMIN.doc/node7.html. The additional keyword required for the use of rotamer moves in basin-hopping is

```
ROTAMERMOVE n1 n2 UNIFORM
! n1 = frequency of rotamer moves
! n2 = number of rotamer moves
```
